# What are the exercise barriers, facilitators and preferences of community-dwelling older adults with heart failure with preserved ejection fraction? A qualitative best fit framework analysis

**DOI:** 10.1136/bmjopen-2024-096413

**Published:** 2025-11-12

**Authors:** Faye Forsyth, Peter Hartley, Jonathan Mant, Scott Rowbotham, John Sharpley, Alison Wood, Christi Deaton

**Affiliations:** 1 Department of Public Health and Primary Care, University of Cambridge School of Clinical Medicine, Cambridge, UK; 2 Nursing Sciences, KU Leuven Biomedical Sciences Group, Leuven, Flanders, Belgium; 3 Primary Care Unit, University of Cambridge, Cambridge, England, UK; 4 Physiotherapy Department, King’s Lynn NHS Foundation Trust, King’s Lynn, Norfolk, UK; 5 Public Health and Primary Care, University of Cambridge School of Clinical Medicine, and Cambridge Biomedical Research Centre Cambridge UK, Cambridge, UK

**Keywords:** Heart failure, Exercise, Patient Satisfaction

## Abstract

**Abstract:**

**Objectives:**

To establish, through patient and public involvement (PPI) events, the exercise barriers, facilitators and preferences of people with heart failure with preserved ejection fraction (HFpEF).

**Design:**

Qualitative ‘best fit’ framework analysis was used to analyse field notes and transcripts collected during three patient and public involvement meetings and three workshops. The best fit framework was based on the **COM-B** model of behaviour change, which has identified that **C**apability, **O**pportunity and **M**otivation components are essential for **B**ehaviour change. The Consolidated criteria for Reporting Qualitative research checklist was used to structure the report.

**Setting and participants**: Community dwelling older adults with HFpEF.

**Results:**

24 people with HFpEF (n=16 female, 66%), 2 spouses and 2 people with chronic conditions participated in the PPI meetings and workshops. Multiple exercise-related capability (negative symptoms, functional ability, resilience and self-efficacy and knowledge and skill); opportunity (appealing components, optimal conditions, adequate support); and motivation factors (well-being, physical gains, goal achievement, sense of enjoyment) were identified as essential to facilitating change in exercise behaviours in people with HFpEF.

**Conclusions:**

This study provides insight into capability, opportunity and motivation conditions that people with HFpEF feel are necessary to enable them to engage in exercise-related behaviour change. This work extends previous post hoc work by moving beyond identification of broad influencers that may enable or impede exercise intervention engagement, to identify intervention conditions necessary to affect change.

**STRENGTHS AND LIMITATIONS OF THIS STUDY**

- The study qualitatively explored the preferences, barriers and facilitators of community dwelling older adults with confirmed heart failure with preserved ejection fraction and their spouses who support them.
- Analysis included the COM-B model as a best-fit framework, which is an evidence-informed model that can identify key capability, opportunity and motivation conditions for behaviour change.
- Use of a predefined model as an interpretive framework could have limited the interpretation of the findings.
- The small sample size limits the generalisability of the results.

## Introduction

Heart failure with preserved ejection fraction (HFpEF) is defined as symptoms of heart failure within the context of a left ventricular ejection fraction ≥50%, structural remodelling and/or diastolic dysfunction plus abnormal biomarkers.^1^ In the most recent update of the global burden of heart failure, Shahim *et al*^2^ highlighted several studies in Europe and North America which demonstrate that the proportion of people with HFpEF is increasing. In older women, where HFpEF is more common,^3^ it accounts for approximately 90% of incident heart failure.^4^ Several factors have been implicated in the rise of cases, including increases in life expectancy, cardiac comorbidities like coronary artery disease and atrial fibrillation and non-cardiac comorbidities like obesity, metabolic disease and diabetes.^5^

Until recently, there were no positive trials of pharmacological therapies in HFpEF,^6^ and management advice focused on treating symptoms and comorbid conditions.^1^ Exercise trials, on the other hand, have a long history of returning favourable results across a broad range of outcomes.^7^ Indeed, consecutive meta-analyses have demonstrated exercise delivers important gains in cardiorespiratory fitness and quality of life in patients with HFpEF.^8^,^10^ Mean improvement in volume of oxygen uptake during peak exercise (VO_2_ peak) in these reviews ranges from 2.28 to 2.6 mL/min/kg. According to Swank *et al*^11^ even modest increases in peak VO_2_ are associated with a favourable outcome. Quality of life improvements from exercise interventions have been more variable. Mean differences in quality of life outcomes between treatment and control arms have ranged from 3.7 points when measured by the Kansas City Cardiomyopathy Questionnaire (not clinically meaningful),^12^ to 10.9 points (clinically meaningful)^13^ in studies employing the Minnesota Living with Heart Failure Questionnaire.^10^ Based on these improvements, exercise training is a Class I recommendation in most national and international guidelines.^1 14 15^ However, multiple gaps in the evidence base remain, including optimal exercise combinations, doses and patient preferences.^7^ This research sought to explore patients with HFpEF’s’ preferences for exercise interventions, and thereby address this gap in the evidence base.

## Background

Exercise intolerance, the cardinal symptom of HFpEF,^1^ causes significant distress and difficulties for patients.^16^ There is consistent evidence that exercise training improves exercise tolerance and functional capacity in patient with heart failure.^17 18^ However, there is a paucity of exercise trials exclusively targeting HFpEF (table 1). Further, completed trials present limitations in their intervention design.^7 18^ For example, the variety of exercises and exercise combinations that have been evaluated is limited. 75% of exercise studies with exclusively HFpEF samples (n=12) have tested conventional aerobic exercises. A very small number of studies (n=2) have evaluated multi-component approaches, and there are few examples of high intensity interval training. Although peripheral non-cardiac mechanisms and oxygen pathway defects are postulated to be pathophysiologically potent in HFpEF,^19 20^ few interventions have assessed alternative types of exercise that have been postulated to better address these causes of exercise intolerance (ie, resistance or inspiratory muscle training).^21^ There are also problems with small sample sizes (mean n=65), mean age of participants (68 years) and clinical status (limited samples include typical multimorbid and frail participants), which raises questions of the generalisability of the results to older individuals. Real-world samples of people with HFpEF indicate a far greater average age.^3^ In the UK, the mean age of the community dwelling OPTIMISE HFpEF cohort was 79 years.^22^ Two contemporary analyses of clinical services indicate a mean age of 80 years^23^ and 78 years,^24^ respectively. Younger samples tend to be healthier with a lower comorbidity burden; a previous review of exercise trials in HFpEF has confirmed the relative lack of comorbid conditions in enrolled subjects.^8^

**Table 1:** Exercise interventions in HFpEF

Author±trial name	Sample	Intervention development work and description of patient involvement in design	Description of intervention components		Category
Gary *et al*^54^	HFpEF	Nil described	Frequency	3× weekly	Aerobic
			Duration	12 weeks	
	n=32 Mean age=67		Description	Warm-up, aerobic training in form of walking, cool-down.	
			Intensity	40–60% of maximal heart rate.	
Wall *et al* 2009^55^	Mixed heart failure	Nil described	Frequency	3× weekly	Aerobic
			Duration	52 weeks	
	n=19 Mean age=69		Description	Warm-up, aerobic treadmill-based exercise, cool-down.	
			Intensity	Guided by symptom severity.	
Kitzman *et al* 2010^56^	HFpEF	Nil described	Frequency	3× weekly	Aerobic
			Duration	16 weeks	
	n=53 Mean age=70		Description	Warm-up, aerobic training walking or cycling, cool-down.	
			Intensity	2 weeks at 40–50% of heart rate reserve, increasing to 60–70%.	
Alves *et al* 2012^57^	Mixed heart failure	Nil described	Frequency	3× weekly	Aerobic
			Duration	6 months	
	n=98 Mean age=62		Description	Warm-up, treadmill/stationary cycle, cool-down.	
			Intensity	70–75% of maximal heart rate.	
Smart *et al* 2012^58^	HFpEF	Nil described	Frequency	3× weekly	Aerobic
			Duration	16 weeks	
	n=98 Age=62.9		Description	Warm-up, aerobic cycle training, cool-down.	
			Intensity	60–70% VO_2_ peak or respiratory rate <30 breaths/minute.	
Kitzman *et al* 2013^59^ SECRET	HFpEF	Nil described	Frequency	3× weekly	Aerobic
			Duration	16 weeks	
	n=63 Mean age=70		Description	Warm-up, aerobic training (walking or cycling), arm ergometry, cool-down.	
Intensity			40–50% of heart rate reserve, increasing to 70%.		
Angadi *et al* 2014^60^	HFpEF	Nil described	Frequency	3× weekly	Aerobic
			Duration	4 weeks	
	n=15 Mean age=69		Description	Warm-up, 2 min intervals of high intensity interval training, cool-down.	
			Intensity	80–85% of maximal heart rate	
Fu *et al* 2016^61^	Mixed heart failure	Nil described	Frequency	3× weekly	Aerobic
			Duration	12 weeks	
	n=129 Mean age=60.5		Description	Warm-up, aerobic interval training, cool-down.	
			Intensity	Intervals at 40% VO_2_ peak and 80% VO_2_ peak.	
Antinocelli *et al* 2016^62^	Mixed heart failure	Nil described	Frequency	3× weekly	Aerobic
			Duration	50 min classes	
	n=343 Mean age=76		Description	Warm-up, cycle ergometer use, cool-down.	
			Intensity	60–70% of predicted maximal heart rate.	
Kitzman *et al* 2016^63^	HFpEF	Nil described	Frequency	3× weekly	Aerobic
			Duration	20 weeks	
SECRET-II	n=100 Mean age=67		Description	Warm-up, walking exercise, cool-down.	
			Intensity	Progressed as tolerated based on heart rate reserve.	
Donelli da Silveira *et al* 2020^64^	HFpEF	Nil described	Frequency	3× weekly	Aerobic
			Duration	12 weeks	
	n=19 Mean age=60.9		Description	High intensity: warm-up, moderate and high intensity aerobic exercise on treadmill, cool-down. Moderate continuous training: warm-up, moderate-intensity aerobic exercise, cool-down.	
			Intensity	High: 80–90% of VO_2_ peak; moderate: 50–60% of VO_2_ peak.	
Lang *et al* 2020^65^	HFpEF	Six-step intervention mapping framework that included two systematic reviews. A PPI group (n=6 with ‘a range of heart failure experiences’, n=3 carers) participated in: design of the interview topic guide and the needs assessment survey, summaries of information from the focus groups.^27^	Frequency	Home-based, encouraged regular activity/use of manual.	Aerobic
			Duration	12 weeks	
	n=50 Mean age=74		Description	Walking programme or chair-based exercise DVD or combination.	
			Intensity	Unclear—tailored to initial fitness assessment.	
Mueller *et al* 2021^66^	HFpEF	Nil described	Frequency	3× weekly high intensity interval training, 5× weekly moderate continuous training.	Aerobic
			Duration	12 months	
OptimEx-Clin	n=180 Mean age=70		Description	HIIT: warm-up, moderate-intensity cycling, high intensity training intervals with active recovery, cool-down. Moderate-intensity continuous training: warm-up, cycle ergometers, cool-down.	
			Intensity	HIIT: 85–95% VO_2_ peak; moderate-intensity continuous training: 50–60% VO_2_ peak.	
Jaarsma *et al* 2021^67^	Mixed heart failure	Longitudinal case study of one patient.^68^	Frequency	5× weekly	Aerobic
			Duration	12 months	
HF-Wii	n=605 Mean age=67		Description	Exergame for 30 min/day	
			Intensity	Not described	
Nilsson *et al* 2008^69^	Mixed heart failure	Nil described	Frequency	2× weekly	Aerobic and resistance
			Duration	16 weeks	
	n=80 Mean age: 69		Description	Warm-up, aerobic dance movements, resistance training, cool-down.	
			Intensity	Aerobic exercises: 15–18 on Borg rating of perceived exertion.	
Davidson *et al* 2010^70^	Mixed heart failure	Nil described	Frequency	1× week	Aerobic and resistance
			Duration	12 weeks	
	n=105 Mean age 71.6		Description	Warm-up, treadmill/stationary cycle, resistance training, cool-down.	
			Intensity	Not described	
Edelmann *et al* 2011^71^	HFpEF	Nil described	Frequency	2× weekly (week 1–4); 3× weekly (week 5–12)	Aerobic and resistance
			Duration	12 weeks	
Ex-DHF-P	n=64 Mean age=65		Description	Warm-up, cycle-based aerobic endurance training, resistance training, cool-down.	
			Intensity	Week 1–4: 50–50% VO_2_ peak. Week 5–12: 70% VO_2_ peak.	
Palau *et al* 2014^72^	HFpEF	Nil described	Frequency	2× daily for 20 min	Inspiratory muscle training
			Duration	12 weeks	
	n=26 Mean age=68		Description	Diaphragmatic breathing	
			Intensity	At a resistance of 25–30% of maximal inspiratory mouth pressure.	
Reeves *et al* 2017^41^	Mixed heart failure	Nil described	Frequency	3× weekly	Aerobic, resistance, balance, mobility
			Duration	12 weeks	
REHAB-HF (pilot)	n=27 Mean age 72.7		Description	Warm-up, balance, mobility, strength and aerobic, cool-down.	
			Intensity	1–2 weeks: <12 on Borg RPE; 13 for endurance and 15–16 for strength training.	
Kitzman *et al* 2021^42^	Mixed heart failure	Pilot trial,^43^ no other development work described.	Frequency	3× weekly	Aerobic, resistance, balance, mobility
			Duration	12 weeks	
REHAB-HF (full trial)	n=349, Mean age=73.1		Description	Warm-up, balance, mobility, strength and aerobic, cool-down.	
			Intensity	1–2 weeks: <12 on Borg rating of perceived exertion; 13 for endurance and 15–16 for strength training.	
Alonso *et al*^28^	Mixed heart failure	Pilot trial with patients with HFrEF only,^73^ no other development work described.	Frequency	Patient directed	Aerobic and resistance
			Duration	18 months	
HEART CAMP	n=204 Mean age=64.6		Description	Warm-up, moderate-intensity continuous training, resistance training, cool-down.	
			Intensity	Continuous training: 40%–80% heart rate reserve. Resistance training: 10–15 repetitions or to fatigue.	
Brubaker *et al* 2023^74^	HFpEF	Previous pilot trials (SECRET-I,^31^ SECTRE-II^63^), no other development work described.	Frequency	3× weekly	Aerobic and resistance
			Duration	70 min	
SECRET-III	n=77 Mean age=67.9		Description	Warm up and cool down (stretching, walking), 40 min aerobic training, 20 min resistance training.	
			Intensity	Individualised and progressive according to baseline exercise test.	

DVD, digital versatile disk; HFpEF, heart failure with preserved ejection fraction; HFrEF, heart failure reduced ejection fraction; HIIT, high intesity interval training; PPI, patient and public involvement; RPE, Rating of pereceived exertion; VO_2_, oxygen uptake.

The mismatch between intervention designs and clinical characteristics of enrolled patients has led some to claim we are ‘fitting a square peg into a round hole’.^25^ There are also concerns about the lack of consideration of HFpEF phenotypes and social determinants of exercise behaviour within trials. As exercise interventions are by their nature complex, key development steps are necessary to ensure the end design takes into account existing evidence on: (1) mechanisms of exercise intolerance; (2) efficacy of exercise modes in addressing these mechanisms; (3) barriers, facilitators and patients’ preferences for operationalisation of exercise interventions.^26^ As table 1 demonstrates, few studies have described undertaking any development work or patient involvement work that satisfies these development pre-requisites, prior to testing their intervention. Even in those programmes that have engaged in development work,^27 28^ there is only one reference to consultation work conducted with people with heart failure and it is unclear whether this sample was comprised of people with HFpEF.^27^

## Aim

The aim of this research was to establish patients with HFpEF patients’ preferences regarding a future exercise intervention.

## Methods

For this analysis, we collated field notes and anonymised transcripts from three patient and public involvement (PPI) meetings (n=15 patients with HFpEF, n=2 with other chronic diseases), and three workshops (n=9 patients with HFpEF, n=2 spouses). Qualitative ‘best fit’ framework analysis was undertaken by an experienced qualitative researcher (FF), supported by specialists in HFpEF (CD) and behaviour change (PH).^29^ The analysis progressed as follows: (1) data sources were imported into NVivo V.12; (2) the best fit framework, based on the COM-B model components, was created within the nodes section; (3) data was coded against the a priori COM-B framework; (4) new themes were created from each node of the framework; (5) the framework, supported by themes and evidence (quotes), formed the basis of this report. Trustworthiness was confirmed by another author (PH) who is familiar with COM-B. They confirmed coded data matched the framework and/or challenged coding decisions; discrepancies were resolved via revisiting the COM-B papers and discussion. Direct quotations have been used to support the theme and framework construct. Pseudonyms have been used and age in decades has been provided to maintain anonymity. Findings are reported in line with the COREQ checklist (Consolidated criteria for Reporting Qualitative research.)^30^

### Sample

Only minimal demographic data (age and sex) were collected as this was a PPI initiative. For workshops, which generated the majority of the data presented, 30 people with a confirmed diagnosis of HFpEF who resided in the East of England were contacted from a completed study, OPTIMISE HFpEF.^31^ Those who responded positively (n=9 people with HFpEF) were asked to sign an informed consent form and cascade information to interested spouses (n=2). People attending PPI meetings (n=15 HFpEF) included some patients with HFpEF who were invited to become a PPI group member during participation in the OPTIMISE HFpEF cohort study,^22 32^ supplemented with members of the National Institute for Health and Care Research Cambridge Biomedical Research Centre, PPI panel.^33^ The latter members (n=2), who had chronic health conditions, had experience of PPI work and were asked to bring this expertise to an otherwise novice PPI group. Meetings and workshops were held in conference style rooms; only workshops were digitally recorded and transcribed verbatim. Meetings and workshops were held between the period 2018 and 2024, meetings lasted 1.5 hours, and workshops were scheduled for 4 hours. All sessions included lunch or refreshments and transportation was arranged; however, there was no reimbursement for time. Rapport had been established via previous participation or regular meetings and communications. Transcripts were not returned to participants for review. There were no topic guides; rather, participants were asked to discuss findings generated from a programme of work seeking to design an optimised diet and exercise intervention in HFpEF^1634^,^37^ and to share their views and suggestions for a future exercise intervention. These findings were presented in PowerPoint format as part of the meetings or workshops, concluding slides had prompts such as ‘what are your views’ and ‘what should we do next’.

### Ethical considerations

Ethical approval to recall participants from the OPTIMISE HFpEF study was granted by the London–Surrey Research Ethics Committee (REC reference: 17/LO/2136.) All participants provided informed consent, including before PPI meetings.

### Data analysis

**Figure 1:**
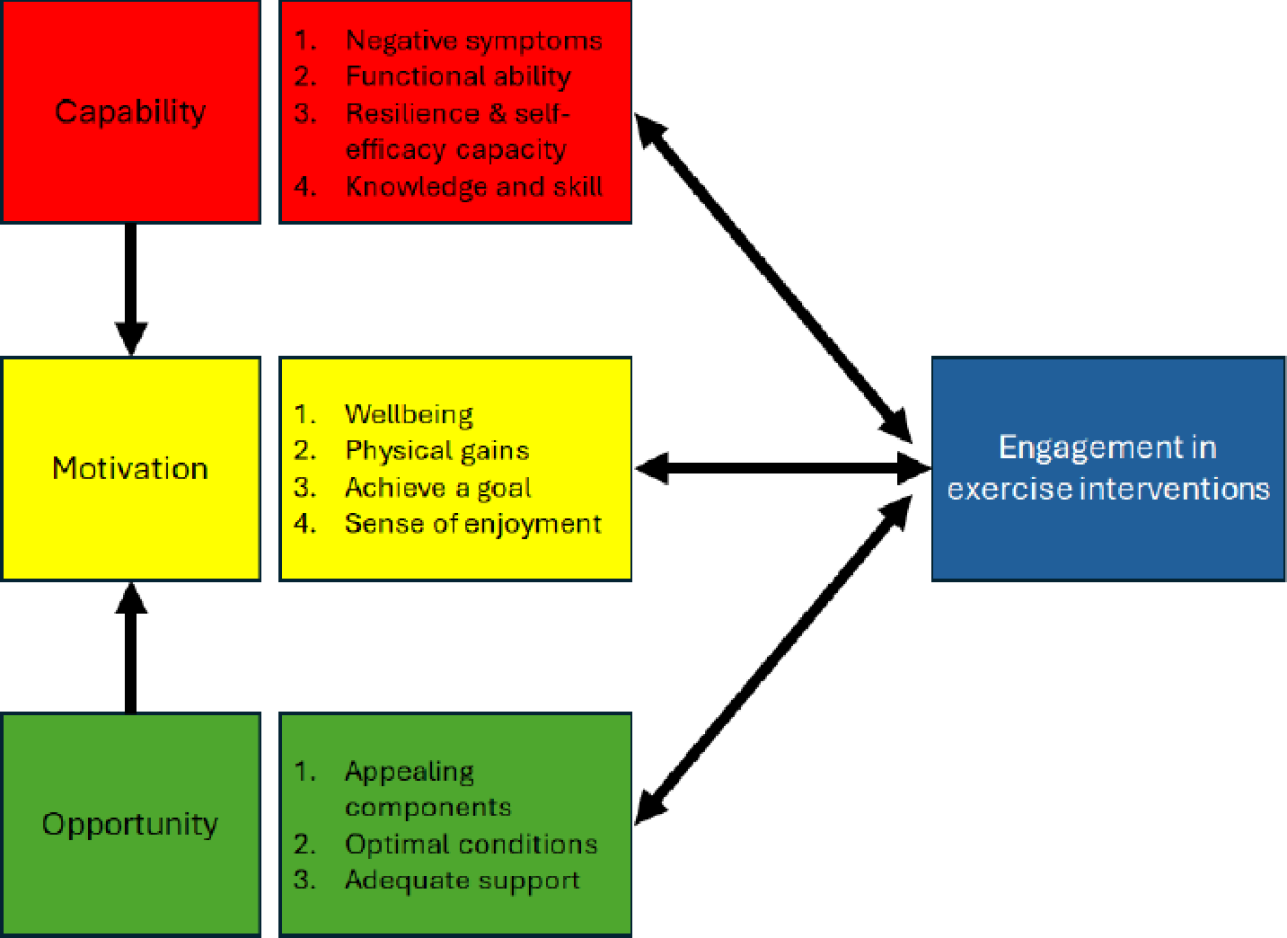
The COM-B behaviour system.

Transcripts and field notes were imported into NVivo V.12 software^38^ for analysis. Qualitative ‘best fit’ framework methods, as described by Dixon-Woods,^29^ were used. This method was employed as it allows for selection of an a priori conceptual framework, in our case the COM-B model for behaviour change,^39^ which then serves as an initial starting point for coding. Having (1) moderated and contributed to all of the meetings and workshops, (2) visited the data regularly over a 6-year period to generate reports and presentations and (3) transcribed recordings or made detailed field notes, we were confident the COM-B model was the best fit for the data. The authors of the COM-B model class it as a ‘behavior system’ which involves three interacting conditions necessary for a behaviour to occur: capability, opportunity and motivation (figure 1).^39^ Within the system, capability is defined as ‘an individual’s psychological and physical capacity to engage in the activity concerned’; motivation as ‘brain processes that energize and direct behaviour’; and opportunity as ‘factors that lie outside the individual that make the behaviour possible or prompt it’. While the authors note that COM-B is a model of behaviour, they stress its utility in designing interventions that intend to change behaviour.^39^ The latter provides further justification for using the COM-B model as a framework for analysis.

### Trustworthiness

Data were coded by one author (FF) and verified by a second author (PH) who co-facilitated the PPI events and who was familiar with the COM-B model through a prior project.^40^ Verification was performed via triangulation of the transcripts with the coded framework and published descriptions of the COM-B model system.

## Results

24 people with HFpEF (n=16 female, 66%, mean age 82 years), 2 spouses and 2 people with chronic conditions participated in this programme of work that included three face-to-face PPI meetings and three workshops. Using the COM-B model of behaviour change, we have identified exercise-related capability, opportunity and motivation conditions that older adults with HFpEF consider essential or preferential to be present, addressed or acknowledged within an intervention designed to elicit behaviour change in this domain. For each COM-B model component, we present a summary of the essential conditions identified and provide quotes in support of these factors. Where multiple factors have been identified, we have prioritised presenting quotes that refer to novel conditions (ie, those not already identified within the HFpEF literature.)

### Exercise capability

To elicit behaviour change, any exercise intervention must acknowledge or address key physical capability and psychological capability factors. Physical capability conditions include: (1) functional ability and (2) negative exercise symptoms. Psychological factors comprise: (1) psychological resilience and self-efficacy and (2) knowledge and skill.

#### Functional ability impacting on capability

According to older adults with HFpEF, functional ability significantly impacts capability to participate in, or attend, exercise interventions. Therefore, this must be accommodated for behaviour change to occur. Age-related deconditioning and post-hospitalisation functional states, which have been identified in previous research,^41^,^43^ were highlighted as important. Novel functional factors included bodily pain. Sources of pain were numerous; however, it frequently included musculoskeletal pain that resulted from falling, joint replacements, oedema and deconditioning.

I had pain in my feet and legs before, but having fallen and getting the pin in my leg, it is very painful. Rosie, 90+ years, female with HFpEF

So if I’m walking… and I have to take a trolley now, only these last two weeks, because my back’s been so bad… I make sure I have some Paracetamols before.” Arlene, 85+years, female with HFpEF

#### Negative symptoms associated with or exacerbated by exercise

Negative symptoms associated with or exacerbated by exercise could be actual or anticipated. Common symptoms of HFpEF like breathlessness and fatigue were frequently cited as problems that must be addressed by, or adjusted for, within an exercise intervention. In most cases, participants perceived that behaviour change could not be achieved in the face of these symptoms, as they either prohibited or limited engagement:

I do get breathless when I exercise so I just stop, …I am often breathless. Colleen, 80+years, female with HFpEF

I just don’t seem to be able to have the energy or be able to get in the right position to be able to do it [exercise]. Derek, 80+years, male with HFpEF

#### Exercise-related psychological resilience and self-efficacy

Participants discussed the importance of having or developing psychological resilience and self-efficacy skills to deal with the stressors associated with exercise such as actual or anticipated symptoms and/or anxiety of these. Fatigue and pain were the most frequently referenced psychological stress triggers or barriers. They required implementation of pre-emptive adaptations for practical, safety or coping reasons. Planning for and overcoming these stressors drew heavily on psychological reserves. Psychological capability to overcome fear of falling was also voiced as an important factor that should be addressed within an intervention to enable participation.

I dread it [exercising], because I know my back’s going to play me up, and I wonder how I’m going to walk back, with difficulty, you know….and that is daunting, really. Arlene, 85+years, female with HFpEF

Previous experience of falling does [affect things], it takes away your confidence, so you are really thinking too hard about what you are doing and it is always there. Rosie, 90+years, female with HFpEF

#### Exercise knowledge and skills

In addition to facilitating psychological resilience, there is the need to provide adequate knowledge and skills to generally exercise correctly and/or to resource patients to exercise safely and effectively in the context of their existing physical capacity issues. Multiple participants were concerned that they may not be performing the exercises in the correct form and/or reported that they could not always follow generic instructions due to problems resulting from pre-existing medical issues.

I think the other thing is when you’re doing it by yourself you might be doing the exercises but you might not be doing them correctly. Martin, 80+years, male with HFpEF

### Exercise opportunity factors necessary for behaviour change

For exercise-related behaviour change to occur, or to enhance the chances of it occurring, participants stated that the ‘right’ opportunities needed to be in place. For this group of older adults with HFpEF and multimorbidity, this meant an intervention that had appealing programme components, that was offered in acceptable conditions and that was accompanied by adequate support. It also meant that certain resources had to be in place or offered for these opportunities to be realised.

#### Appealing programme components

While there was some variability, there was broad consensus among participants that exercise interventions should be enjoyable, fun or social. Those endorsing social programmes preferred group-based exercises. Other programme characteristics deemed essential were content that was: gradually built up; challenging; varied; that could be maintained, extended or routinised into home life; and which had embedded targets and goals.

I do it [exercise]partly because it keeps all the bits of your body moving and partly because it is very social. Maeve, 75+years, female with HFpEF

#### Optimal programme conditions for exercise

To increase the likelihood of people with HFpEF attending or adhering to an exercise intervention and thus affecting behaviour change, participants suggested optimal programme conditions. These included sessions that were provided at an appropriate time and in a convenient place that was of an adequate size to accommodate freedom of movement and equipment for mobilising. Other conditions that were considered essential were flexibility and an indefinite duration.

As long as it’s not in the evening, because unless it’s in [local area]and I could try and walk, but there again I don’t like to do walking out at nine o’clock at night back home. Arlene, 85+years, female with HFpEF

#### Adequate support for exercising

Some participants stressed the importance of having knowledgeable instructors whom they trusted and could access for feedback. For others, support to personalise programmes or information to reinforce any new knowledge and skills was essential to drive behaviour change. Adequate support also encompassed provision of resources that would help people with HFpEF realise any opportunities. This included financial and travel support, as well as assistance to alleviate people from care duties.

That [transportation] is for me now problematic, because if I go, costs me £15 there, £15 back….Twice a week, £60 a week. Graham, 85+years, male with HFpEF

### Motivations for engaging in an exercise intervention

Participants spoke of their aspiration that an exercise intervention would lead to them feeling better or happier, or that it would result in an increase in general well-being. Specific motivators included regaining physical ability, seeing results or a future result trajectory, achieving a goal, or a sense of achievement/enjoyment.

Well a lot of my exercise is to do with the garden, and I do absolutely enjoy that, because I can see results, so to see a result of some sort, especially if it’s on the plus side, then you’re gaining two-fold, you’ve enjoyed doing it, and then you’ve seen the results. Arlene 85+years, female with HFpEF

### Discussion

To our knowledge, this is the first study to prospectively explore what intervention conditions people with HFpEF consider essential to affect behaviour change in the domain of exercise (figure 1). Multiple behaviour change challenges were noted and strategies to address these within any future exercise intervention would be needed. This research has also identified ‘optimal’ conditions which people with HFpEF consider essential to enable exercise-related behaviour change. Reflecting back to the list of exercise interventions summarised in table 1, it would seem that few exercise interventions evaluated to date have undertaken development or PPI work that would help ensure the end intervention is cognisant of the conditions people with HFpEF consider essential for behaviour change. Further, based on our review of their reported outcome measures, it does not appear as though any intervention thus far tested has aimed to assess outcomes that people with HFpEF consider essential (well-being or enjoyment). While we accept that many of these studies were mechanistic or proof of concept, there is sufficient evidence of efficacy to be able to extend research programmes beyond proving exercise has a role in managing HFpEF. Arguably, all interventions should be accompanied by process evaluations, so that we can start to improve intervention design and implementation.

Participants were clear that they had significant functional capacity issues relating to exercise performance and experienced multiple negative symptoms on exercising, that must be accommodated and addressed within an intervention designed to change behaviour patterns. In other words, an intervention that focuses only on delivering a programme of exercise without building in strategies to screen, assess and manage physical inability, pain, breathlessness or fatigue could result in an intervention that people with HFpEF feel incapable of engaging with. There is emerging evidence confirming the importance of distinct mechanisms of exercise intolerance in HFpEF, that are based on phenotypic presentation.^44^ It is possible that phenotypic evaluation coupled with an assessment of patients’ descriptions of barriers and preferences could be simultaneously undertaken, thus enabling personalised approaches. Participants also stressed the need for knowledge and skills (what, why, how) to exercise safely and effectively and psychological resilience and self-efficacy capacity to overcome psychological barriers like fear, low confidence and anticipated symptoms. Any intervention must address these capability conditions to be effective; however, they must equally be mindful to embed these strategies within the right opportunities to make the execution of a new behaviour possible. In this respect, people with HFpEF have varied opinions of what is ‘right’ for them, be it programme components, conditions of delivery or support. However, there was broad agreement that overall, any exercise intervention should be enjoyable, fun and social. Lastly, interventions must ensure they are tapping into motivators that embed behaviour change, which might be achieved by goal setting and regular reflection or simple well-being scales. Previous research has demonstrated that adherence can be difficult to achieve in heart failure;^45^ therefore, motivators are of particular importance.

#### Comparison with other literature

We believe this is the first qualitative report to describe capability, opportunity and motivation conditions for exercise behaviour change, as expressed by people with HFpEF. However, we were able to retrieve one abstract and two reports that offer relevant comparisons. De Wilde *et al*^46^ assessed barriers to exercise and self-efficacy using a survey methodology in 60 patients with HFpEF participating in the PRIORITY (PeRsonalIzed remOtely guided preventive exeRcIse therapy for a healThY heart) randomised controlled trial.^47^ The authors reported that most participants (50%) would not engage with an exercise programme if they perceived it to be boring or not fun; or if they had to exercise alone (40%). They also indicated that a good instructor was essential and that there must be flexibility in a programme to accommodate life events. When they compared different severities of HFpEF (ie, Stage A vs B vs C), they found that those with more severe symptomology (Stage C) were less confident that they could overcome resource and symptom-related barriers to engage in exercise.

Smith *et al*^48^ interviewed 15 patients and 7 carers following participation in the REACH-HFpEF intervention, which included a DVD led, chair-based exercise component. They determined that personalised exercise support, goal setting and feedback enhanced engagement, motivation and self-efficacy. Those engaging well reported improved social, emotional and physical outcomes from participation. Concurrent multimorbidity, functional capacity issues and limited perceived gains were reported to reduce or restrict participation. Salahshurian *et al*^49^ analysed 67 interviews, conducted longitudinally throughout an exercise intervention (HEART Camp), to establish factors that affected adherence. Important adherence barriers included low functional capacity and negative emotional states. These influenced decisions to continue or cease participation via motivation pathways. Those continuing in the programme reported multiple improvements across a range of domains, not necessarily specific to their heart failure. The interviewers also established critical recommendations that patients with HFpEF would give to other patients embarking on a similar endeavour such as establishing routines, finding ways to make it enjoyable and tracking down the optimal conditions that made exercise easy to action.

There are more reports that have looked at perceptions of exercise,^50^ exercise influencers,^51^ exercise barriers and enablers^52^ and long-term adherence^53^ in unspecified heart failure or heart failure with reduced ejection fraction (HFrEF). Albert *et al*^50^ interviewed 48 patients with HFrEF who took part in the HF-ACTION trial and determined that participants reported a lack of knowledge or access to information and that if accessed, information provided was often not meaningful. They also reported that fear was a driver of action and inaction, life often got in the way of being active and emotions and perceptions of capability played a role in regulating activity. Tierney *et al*^51^ interviewed 22 people with presumed HFrEF and reported that cognitive, emotional, interpersonal and environmental factors played a role in willingness to exercise. Within these categories, mental outlook, fluctuating health, environmental influences and others’ expectations were key in enabling or inhibiting engagement in exercise. Warehime *et al*^53^ interviewed 22 participants about long-term adherence to an exercise programme. Important adherence enablers identified within interviews included having sufficient knowledge, support from trainers and family and adequate motivation. In the most recent analysis, Amirova *et al*^52^ used the Theoretical Domains Framework,^39^ which complements the COM-B model, to identify perceived barriers and enablers to exercise in patients with HFrEF (n=16). They elicited 39 belief statements about exercise that functioned as barriers of facilitators. Most commonly expressed inhibitors were beliefs about physical ability, low self-efficacy, lack of social support and concern about adverse events. Facilitators included goal setting and action planning. There are multiple overlaps between themes from these investigations and the data presented here, particularly around capacity, knowledge, skills and motivation. However, these studies limit their remit to broad suggestions of how these factors may influence activity. They did not explore how these influencers may be addressed through study design as we have done.

### Study limitations

This study is limited by the small sample size and reliance on field notes for some data which may introduce bias. Two participants did not have HFpEF, and were co-opted on the basis of their PPI experience; this may have influenced results. The study is further limited by the lack of data on broader psychosocial and biological factors that may influence exercise engagement. While the sample was representative in terms of established characteristics of HFpEF (older, predominantly female, multimorbid), it is constrained by the fact that participants were recruited from a single site in the East of England. Further bias may be introduced through the use of workshops, which are akin to focus groups in that there is a risk of social acceptability bias. However, the diversity of the responses and the concordance with the breadth of findings from other studies suggests that the risk of social desirability bias is low.

### Conclusions

Findings from this study provide insight into capability, opportunity and motivation conditions that are necessary to enable people with HFpEF to change exercise behaviours. This work extends previous research by moving beyond establishing broad influences that may enable or impede behaviour change to identify intervention conditions necessary to affect change. In identifying these conditions, participants have also elucidated potential methods through which these behaviour change conditions can be met. For example, future interventions might wish to offer group classes that build in social components that make the experience fun and enjoyable. Further, this research indicates that additional outcome measures will be needed to capture the type of changes that motivate people with HFpEF to execute behaviour change. These measures could in turn help people with HFpEF reflect on the benefits of performing any new behaviours (which might not be immediately obvious or recalled), thus changing the behaviour from something they must do as part of an intervention, to something they want to do. We know that exercise is efficacious in HFpEF. Ongoing research to identify the optimal modes of exercise, combined with larger explorations of patient preferences and process evaluations of interventions tested, is vital to moving the field forward.

## Data Availability

Data are available upon reasonable request.
