# Supplementary material for: What are the exercise barriers, facilitators and preferences of community-dwelling older adults with heart failure with preserved ejection fraction? A qualitative best fit framework analysis
Source: BMJ Open. 2025 Nov 12;15(11):e096413. doi: 10.1136/bmjopen-2024-096413 (PMC12612770; doi:10.1136/bmjopen-2024-096413)
Supplement: Supplementary file 1 [file bmjopen-2024-096413.reviewer_comments.pdf]

PEER REVIEW HISTORY

BMJ Open publishes all reviews undertaken for accepted manuscripts. Reviewers are asked to complete a checklist review form and are provided with free text boxes to elaborate on their assessment. These free text comments are reproduced below.

ARTICLE DETAILS

Title (Provisional)

What are the exercise barriers, facilitators and preferences of community dwelling older adults with Heart Failure with preserved Ejection Fraction: a qualitative best fit framework analysis.

Authors

Forsyth, Faye; Hartley, Peter; Mant, Jonathan; Rowbotham, Scott; Sharpley, John; Wood, Alison; Deaton, Christi

---

VERSION 1 - REVIEW

**Reviewer**	**1**
**Name**	**Upadhya, Bharathi**
**Affiliation**	**Duke University School of Medicine, Cardiovascular Medicine**
**Date**	**26-Dec-2024**
**COI**	

This study is well done and addresses the major unmet needs in HFpEF.

I do not have any major concerns.

Few suggestions.

Page 6 – Please include the following

RCTs do not account for frailty, multiple comorbidities, and lack of data on high-intensity interval training resistance training early after acute decompensated HF. RCTs do not address the social determinants and heterogeneity of HFPEF /HFpEF clinical phenotypes for ET.

Discuss future goals

• Although most attention is focused on psychosocial/behavioral factors, it is conceivable that biological factors may also help determine whether individuals maintain an exercise program once initiated. There are studies of biological predictors of physical activity behavior, including genetic markers. Lancet. 2012;380:258–271.

• Better phenotype the predominant mechanism of exercise intolerance (effects of obesity, sarcopenia, chronotropic incompetence, impaired peripheral vascular responses, etc) – then developing ET specific to that.

• Optimize exercise training regimens through better tailoring to different types of patients (Frailty, significantly older adults, multi-comorbidities, social determinants, cognition, mental health, financial resources) -------- Variables to consider include exercise mode, program duration, frequency, and intensity, and use of novel training techniques (high-intensity intervals, prolonged sessions, optimal mix of aerobic, resistance, balance, flexibility). Other variables include the potential safety and efficacy of beginning training early after acute decompensated HF or during hospitalization.

• The major clinical hindrance to the use of ET as a therapeutic option in the HF population is how to get individuals to initiate and maintain an ET program. Despite a well-organized and resourced effort to optimize adherence in HF-ACTION, only ~40% of patients in the intervention arm achieved the target of 90 minutes of exercise/week at 3 months - developing educational and motivational tools, user-engaging, and personalized training programs.

**Reviewer**	**2**
**Name**	**Griffith, Garett**
**Affiliation**	**Northwestern University, Northwestern University**
**Date**	**20-Feb-2025**
**COI**	

The authors provide a qualitative assessment describing preferences of HFpEF patients related to an exercise intervention. While this study represents an important potential contribution to this body of literature, there are several clarifications that are required to address this topic more strongly:

1) In the abstract, define “PPI” before using this acronym for the first time.

2) In the “What is already known on this topic” – it would be helpful to be more specific regarding which outcomes are impacted by exercise in this group. Is this in reference to quality of life, fitness, prognostic outcomes, and/or something else?

3) “Data” are plural – please correct subject/verb agreement throughout accordingly.

4) It is stated that sex and age were the only demographic data collected/reported for this study. Does the study team have any clinical information available for these patients? Generalizability would be improved if the study confirmed clinical features of the patients represented in these qualitative findings.

5) What additional models of exercise need to be explored in this population (for instance, home-based, telemedicine, etc.)? The authors might consider expanding the discussion and recommendations for next steps in this area of research accordingly.

**Reviewer**	**3**
**Name**	**Baik, Dawon**
**Affiliation**	**University of Colorado**
**Date**	**01-Aug-2025**
**COI**	

I appreciate the opportunity to review this manuscript. My comments are as follows:

• This study aimed to establish HFpEF patients' preferences regarding a future exercise intervention. However, it is unclear why two participants with other chronic conditions and two spouses were included. Their inclusion may have influenced the findings and should be clearly justified, including how the researchers accounted for the perspectives and suggestions of participants who did not have HFpEF.

• Participant input was collected from three PPI meetings and two workshops, but the distinction between the two formats is unclear. Clarification on how the meetings and workshops differed in structure, purpose, or content would strengthen the methodology.

• The PPI meetings appear to resemble exit interviews conducted after the OPTIMISE HFpEF project. A brief description of the OPTIMISE HFpEF project would provide helpful context.

• The authors stated that no topic guides were used during the meetings. It is unclear what the specific purpose of the PPI meetings and workshop was. Without a defined objective or the use of prompts or guiding questions, it is difficult to understand how participants' views and suggestions were effectively elicited and incorporated.

• The authors stated that data were collected through three PPI meetings and three workshops; however, important details are missing. Specifically, how many participants attended each meeting or workshop? Where were these sessions held? How long did each session last? Were participants compensated? Additionally, when did data collection begin and end?

• The description of the qualitative data analysis lacks sufficient detail. More information is needed regarding how the transcripts were coded, how categories, subthemes, and themes were developed, and how discrepancies between coders were resolved. The methods used to ensure trustworthiness also appear to be weak and should be strengthened.

• The Results section should include information on the mean age of participants, as age data were collected.

• The results do not seem fully aligned with the study's stated aim. While the findings primarily address facilitators and barriers encountered by participants following an exercise intervention, the study's objective was to explore HFpEF patients' preferences regarding optimal exercise intervention combinations. I recommend reframing the aim and corresponding findings for better coherence.

• In the Discussion, clearly highlight the novel findings and explicitly explain how this study contributes to the existing body of knowledge.

VERSION 1 - AUTHOR RESPONSE

Reviewer 1's Comments	Authors' Response
This study is well done and addresses the major unmet needs in HFpEF.<br>I do not have any major concerns.	
Few suggestions. Page 6 – Please include the following RCTs do not account for frailty, multiple comorbidities, and lack of data on high-intensity interval training resistance training early after acute decompensated HF. RCTs do not address the social determinants and heterogeneity of HFPEF /HFpEF clinical phenotypes for ET.	
Discuss future goals<br><br>• Although most attention is focused on psychosocial/behavioral factors, it is conceivable that biological factors may also help determine whether individuals maintain an exercise program once initiated. There are studies of biological predictors of physical activity behavior, including genetic markers. Lancet. 2012;380:258–271.	
• Better phenotype the predominant mechanism of exercise intolerance (effects of obesity, sarcopenia, chronotropic incompetence, impaired peripheral vascular responses, etc) – then developing ET specific to that.	
• Optimize exercise training regimens through better tailoring to different types of patients (Frailty,	

significantly older adults, multi-comorbidities, social determinants, cognition, mental health, financial resources) -------- Variables to consider include exercise mode, program duration, frequency, and intensity, and use of novel training techniques (high-intensity intervals, prolonged sessions, optimal mix of aerobic, resistance, balance, flexibility). Other variables include the potential safety and efficacy of beginning training early after acute decompensated HF or during hospitalization.	
• The major clinical hindrance to the use of ET as a therapeutic option in the HF population is how to get individuals to initiate and maintain an ET program. Despite a well-organized and resourced effort to optimize adherence in HF-ACTION, only ~40% of patients in the intervention arm achieved the target of 90 minutes of exercise/week at 3 months - developing educational and motivational tools, user-engaging, and personalized training programs.	

**Reviewer 2's Comments**	**Authors' Response**
The authors provide a qualitative assessment describing preferences of HFpEF patients related to an exercise intervention. While this study represents an important potential contribution to this body of literature, there are several clarifications that are required to address this topic more strongly:.	
1) In the abstract, define "PPI" before using this acronym for the first time	
2) In the "What is already known on this topic" – it would be helpful to be more specific regarding which outcomes are impacted by exercise in this group. Is this in reference to quality of life, fitness, prognostic outcomes, and/or something else?	

3) "Data" are plural – please correct subject/verb agreement throughout accordingly.	
4) It is stated that sex and age were the only demographic data collected/reported for this study. Does the study team have any clinical information available for these patients? Generalizability would be improved if the study confirmed clinical features of the patients represented in these qualitative findings.	
5) What additional models of exercise need to be explored in this population (for instance, home-based, telemedicine, etc.)? The authors might consider expanding the discussion and recommendations for next steps in this area of research accordingly.	

**Reviewer 3's Comments**	**Authors' Response**
• This study aimed to establish HFpEF patients' preferences regarding a future exercise intervention. However, it is unclear why two participants with other chronic conditions and two spouses were included. Their inclusion may have influenced the findings and should be clearly justified, including how the researchers accounted for the perspectives and suggestions of participants who did not have HFpEF.	
• Participant input was collected from three PPI meetings and two workshops, but the distinction between the two formats is unclear. Clarification on how the meetings and workshops differed in structure, purpose, or content would strengthen the methodology.	
• The PPI meetings appear to resemble exit interviews conducted after the OPTIMISE HFpEF project. A brief description of the OPTIMISE HFpEF project would provide helpful context.	

• The authors stated that no topic guides were used during the meetings. It is unclear what the specific purpose of the PPI meetings and workshop was. Without a defined objective or the use of prompts or guiding questions, it is difficult to understand how participants' views and suggestions were effectively elicited and incorporated.	
• The authors stated that data were collected through three PPI meetings and three workshops; however, important details are missing. Specifically, how many participants attended each meeting or workshop? Where were these sessions held? How long did each session last? Were participants compensated? Additionally, when did data collection begin and end?	
• The Results section should include information on the mean age of participants, as age data were collected.	
• The description of the qualitative data analysis lacks sufficient detail. More information is needed regarding how the transcripts were coded, how categories, subthemes, and themes were developed, and how discrepancies between coders were resolved. The methods used to ensure trustworthiness also appear to be weak and should be strengthened.	
• The results do not seem fully aligned with the study's stated aim. While the findings primarily address facilitators and barriers encountered by participants following an exercise intervention, the study's objective was to explore HFpEF patients' preferences regarding optimal exercise intervention combinations. I recommend reframing the aim and corresponding findings for better coherence.	
• In the Discussion, clearly highlight the novel findings and explicitly explain how this study contributes to the existing body of knowledge.	
